# Palliative Care: Too Good to Be True?

**DOI:** 10.5041/RMMJ.10394

**Published:** 2020-10-14

**Authors:** Helen Senderovich, Kristen McFadyen

**Affiliations:** 1 Geriatrics & Palliative Care & Pain Medicine, Baycrest Health Sciences, Toronto, Ontario, Canada; 2 Assistant Professor of the University of Toronto, Department of Family and Community Medicine, Division of Palliative Care, Toronto, Ontario, Canada; 3 Faculty of Medicine, University of Toronto, Toronto, Ontario, Canada

**Keywords:** Barriers to palliative care, integration, model of palliative care, multidiscipline care coordination, palliative care

## Abstract

**Introduction:**

Many patients and their families are hesitant to consult a palliative care (PC) team. In 2014, approximately 6,000,000 people in the United States could benefit from PC, and this number is expected to increase over the next 25 years.

**Objectives:**

The purpose of this review is to shed light on the significance of PC and provide a holistic view outlining both the benefits and existing barriers.

**Methods:**

A literature search was conducted using MEDLINE (PubMed), Cochrane Central Register of Controlled Trials, and Web of Science to identify articles published in journals from 1948 to 2019. A narrative approach was used to search the grey literature.

**Discussion:**

Traditionally, the philosophy behind PC was based on alleviating suffering associated with terminal illnesses; PC was recommended only after other treatment options had been exhausted. However, the tenets of PC are applicable to anyone with a life-threatening illness as it is beneficial in conjunction with traditional treatments. It is now recognized that PC services are valuable when initiated alongside disease-modifying therapy early in the disease course. Studies have shown that PC decreased total symptom burden, reduced hospitalizations, and enabled patients to remain safely at home.

**Conclusion:**

As the population ages and chronic illnesses become more widespread, there continues to be a growing need for PC programs. The importance of PC should not be overlooked despite existing barriers such as the lack of professional training and the cost of implementation. Education and open discussion play essential roles in the successful early integration of PC.

## INTRODUCTION

Palliative care (PC) is a specialized area of health care that aims to improve patient quality of life through expert symptom monitoring, psychosocial care, patient–physician communication, facilitation of treatment planning, and comprehensive discussions with family members with respect to end-of-life care planning. A key to delivering PC is incorporating a multidisciplinary team and comprehensive approach which addresses not only the physical aspect, but also the emotional, spiritual, and practical domains. Over the last few decades, there has been rising awareness regarding the benefits of integrating PC with curative interventions. Unresolved questions as well as barriers remain, regarding how to successfully promote the integration of PC.

Palliative care is a relatively new medical subspecialty which has evolved substantially during the last six decades. It originated as hospice care in the late 1940s, specifically aimed at improving the quality of life of patients with terminal illnesses. Growing dissatisfaction and concern with the care of terminally ill patients, due to a preoccupation with curative interventions, led to an increased focus on quality of life. In 1990, the World Health Organization (WHO) recognized PC as a distinct specialty, defined as:

[an] approach that improves the quality of life of patients and their families facing the problems associated with life-threatening illness, through the prevention and relief of suffering by means of early identification and impeccable assessment and treatment of pain and other problems, physical, psychosocial and spiritual.^1^

Integration of PC was first introduced in Canada in 1999 under the Palliative Care Integration Project (PCIP). In 2005, the End of Life Care Strategy (EoLCS) further expanded the implementation of PC, including integration, coordination, and quality of care.^2^

In 2011, over 29 million people died from terminal illnesses globally, and it is estimated that 20.4 million people require PC at the end of their lives each year.^1^ A study by Murtagh showed that 69%–82% of patients who died in high-income countries could have benefited from a PC program.^3^ The majority of adults in need of PC died from cardiovascular diseases (39%), cancer (34%), and chronic respiratory diseases (10%).^1^ In terms of the global distribution of the need for PC, 78% of adults requiring PC at the end of life are located in low- and middle-income countries; however, the largest rate per 100,000 adult population in need of PC comes from higher-income countries.^1^ Currently, only 14% of those who need PC are receiving it worldwide.^4^ Thus, only a fraction of people who require PC are receiving it, and with the increasing number of complex comorbidities and the growing aging population, there continues to be a large unmet need for PC programs.

Despite the value of PC, significant barriers remain to integrating PC services into existing healthcare systems. The WHO emphasizes a public health model with a focus on policy, education, medication availability, and implementation for PC development^1^; however, a number of barriers exist to achieving these components. For instance, many countries lack government support and policies that support the provision of PC. Without policies and funding mechanisms in place for PC development, the growth of PC is limited to whatever can be achieved by community and clinical leaders. Additionally, the majority of healthcare professionals receive little or no training on the principles and practice of PC. The availability of PC medications varies greatly worldwide, with 80% of the worldwide population lacking adequate access to opioids.^1^ Lastly, psychological factors and access to financial resources can limit access to PC in many countries.^1^

There have been great strides in illuminating the need for PC among patients with terminal illnesses. For instance, patients with end-stage renal disease (ESRD) experience high rates of chronic pain, depression, cognitive impairment, and a reduced life expectancy. Although PC is associated with improved symptom management and reduced healthcare costs, it is underutilized by this patient population due to high healthcare costs and lack of provider training.^5^ This leads to late referral to PC services, and ultimately late integration of PC. The large unmet need to alleviate the suffering of patients with ESRD highlights an area for expansion of PC programs.^5^ Studies show recent expansion of PC, with over 1,000 new hospital-based PC programs created in the United States over the past 10 years.^5^ Globally, PC has been applied for symptomatic relief in patients with respiratory failure due to drug-resistant tuberculosis, emphasizing the value of PC used in conjunction with active disease treatment.^1^ Palliative care is considered to be an integral part of a comprehensive care program, and its importance is also seen in developing countries. For example, in Qatar, PC was established in 2008 as a focus of education, research, and treatment.^7^ The progressive shift towards integrating PC alongside curative interventions in Western countries for patients with advanced and serious illnesses further emphasizes the need for improved access to PC programs.^1^

Despite the clear benefits of PC, many patients and their families remain hesitant to initiate a consultation with a PC team.^8^ Lack of public awareness regarding what PC is and what it can and cannot do, along with the associated stigma which equates PC with death, may result in a reluctance to access PC in a timely manner. The primary goal of this review was to examine the current PC literature and provide a holistic perspective that outlines both the benefits of PC and ongoing barriers to its implementation.

## METHODS

### Search Strategy

This structured narrative review was planned and conducted according to the guidelines in the Preferred Reporting Items for Systematic Reviews and Meta-Analysis (PRISMA), in conjunction with a more narrative approach (especially with respect to grey literature). English-language literature was evaluated by searching PubMed, the Cochrane Central Register of Controlled Trials, and Web of Science (1948 to 2019) for the terms: “palliative care,” “multidiscipline approach,” “care coordination,” “existing integrated model of palliative care,” “early referral of the palliative care,” and “existing barriers of palliative care.” Grey literature was searched using the metaRegister of controlled trials (active and archived registers; May 2018). Identified studies were tracked using Scopus. The identified studies and review articles were verified to discover further relevant studies and avoid any unintentional exclusions.

### Inclusion and Exclusion Criteria

Screening criteria were established for the published content and study type of the located literature (Table 1).

**Table 1:** Screening Guidelines Used to Locate Appropriate Literature for this Review.

Guideline	Include	Exclude
Does this study describe the important aspects of palliative care?	Palliative care is defined as follows: Patient- and family-centered care that optimizes quality of life by anticipating, preventing, and treating suffering Palliative care may be reported as: End-of-life care Restorative care Bereavement care	Experimental studies that do not evaluate palliative care interventions Experimental studies evaluating palliative care not focused on patient–family dyad
What type of study is it?	Randomized controlled trials Cross-sectional studies Cohort studies Observational studies Controlled before–after studies Interrupted time series studies Repeated measure studies Non-randomized trials Longitudinal studies	Commentaries Case reports Case series Editorials

Once publications were screened, the inclusion and exclusion criteria detailed in Table 2 were applied.

**Table 2:** Literature Inclusion and Exclusion Criteria.

Inclusion Criteria	Exclusion Criteria
Human studies published in English Peer-reviewed observational and experimental studies (i.e. no commentaries) Studies that report the existing model of PC Studies involving palliative care at any level, including practicing specialists, general practitioners, patients, and/or family members Studies focusing mainly on existing PC barriers Qualitative and quantitative research studies	Experimental studies not evaluating palliative care interventions Experimental studies evaluating palliative care but not focused on patient–family dyad Non-English-language studies Animal studies

## RESULTS

An initial database search yielded 189 studies. Two reviewers (HS, EL) used Covidence (Veritas Health Innovation, Melbourne, Australia) to independently assess whether the primary studies met the inclusion criteria. Duplicate entries were identified and removed, and the selection of articles was finalized by consensus. An additional 111 papers were excluded as they did not meet the inclusion review criteria, leaving 78 publications for further assessment. The investigators further reviewed the reference lists from these articles and grey literature. A total of 23 articles were identified as duplicates and also excluded from the review. A total of 55 articles were selected on the basis of the eligibility criteria; 3 additional articles deemed pertinent to the focus of this review were added, giving a final total of 58 articles reviewed.

## DISCUSSION

The goals of PC (Box 1) revolve around the prevention and alleviation of pain and discomfort associated with life-threatening illnesses; care plans are developed in close collaboration with patients and their families. This ensures that care aligns with the patient’s values and preferences. Consistent and sustained communication between the patient and caregivers is vital in providing support to the patient and their family. Clearly communicating diagnosis, prognosis, and what to expect in the future improves satisfaction with care.^9^ Studies have shown that PC relieves the burden on family members and improves patient quality of life.^10^,^11^

Box 1 **Goals of Palliative Care**

✓ Control pain and other symptoms
✓ Relieve burden on family members
✓ Improve understanding of the illness and what to expect in the future
✓ Address physical, social, psychosocial, and spiritual domains of care
✓ Evaluate treatment alternatives in the context of patients’ goals and values
✓ Improve patients’ quality of life

### Benefits of Palliative Care

The many benefits of utilizing PC approaches are clear and have been highlighted by various randomized control trials (RCTs). Brännström and Boman^12^ used a sample of 62 congestive heart failure (CHF) patients with New York Heart Association (NYHA) class III/IV symptoms to compare disease-modifying treatment to palliative home care, also known as the “PREFER” model. Patients who were randomly chosen to receive PC experienced an improved health-related quality of life, greater symptom relief, and fewer re-hospitalizations.^12^ The incorporation of a PREFER model ultimately led to decreased morbidity and total symptom improvement. Additionally, a study by Sidebottom et al. compared PC to curative inpatient care and showed that those who received a PC referral had a short-term improvement in total symptom burden, quality of life, and depressive symptoms at 1 and 3 months follow-up.^13^ Wong et al. published a phase-three RCT of 84 patients with NYHA class III/IV symptoms admitted to hospital with decompensated CHF, which showed a 55% reduction in readmission rate at 12 weeks in patients in the multidisciplinary PC program.^14^ More recently, a single-center RCT conducted by Rogers et al., which looked at the combination of PC interventions during hospitalization, reported significant improvements in quality of life at 6 months after discharge in an outpatient environment.^15^ Palliative care can also reduce healthcare costs. This is based on the concept that if the patient is being well cared for, there is an expectation of fewer complications and fewer unnecessary hospital visits.^16^ For example, a mobile integrated healthcare (MIH) program that was held over the course of 6 months in Florida produced a net saving of over US$2.4 million.^17^ The MIH is a delivery model that provides care on an as-needed basis and aims to prevent any gaps in the healthcare services. Closing these gaps will decrease healthcare costs by reducing the number of emergency hospital visits, thereby leading to an improved patient experience. Member satisfaction was also scored as very high, with 86% of respondents saying that they would recommend the program to friends and family.^17^ Overall, a multidisciplinary PC program is associated with improvements in patient quality of life, symptom burden, advance care planning, patient and caregiver satisfaction, and reduced healthcare utilization.^18^

Traditionally, the philosophy of PC was based on alleviating suffering associated with terminal illnesses, and it was utilized as comfort care once curative treatment options were exhausted.^19^ A 2016 survey, conducted as part of the HoldFAST study, found that healthcare providers would only refer heart failure patients to PC when they had nothing further to offer.^20^ This highlights the need for improved awareness regarding the role and goals of PC within both the medical community and the general public. The tenets of PC may be applicable to anyone with a life-threatening illness and can provide benefit when offered in conjunction with curative treatment options at all stages of disease. Studies show that the provision of a PC program should be considered based on need and not diagnosis since there is no apparent difference between patients with non-malignant life-limiting chronic illness and patients with cancer.^21^

Previously, a strict dichotomy existed, where patients initially received curative care until it failed, and then switched to PC.^22^ This model emphasized an abrupt shift to PC when no further attempts at disease-modifying treatment are feasible. Recently, there has been a switch towards an overlapping model of PC where patients receive a gradually increasing degree of PC while less curative/restorative care was received.^22^ The newly accepted model of PC places greater emphasis on the introduction of PC earlier during the disease trajectory. This integrated model of PC focuses on providing PC concurrently with curative treatment, beginning at the time of patient admission. Both disease-modifying/curative and palliative modalities are used simultaneously.^22^ Applying PC at all stages of an illness requires continual reassessment of physical exam findings, symptom assessment scales, laboratory findings, psychological evaluation, and patient level of comfort to achieve optimal symptom management.^22^ It is important to note that this model of integrative care continues after death in order to address the bereavement needs of the patient’s family, in contrast to curative therapy, which ends prior to death.

It has been widely acknowledged that patients with non-malignant chronic illness, such as those with chronic obstructive pulmonary disease (COPD), benefit from access to PC.^21^ Additionally, a 2010 study conducted at Massachusetts General Hospital in Boston revealed that patients with lung cancer who received PC integrated with standard cancer care experienced less depressive symptoms (16% versus 38%, *P*=0.01) and rated a better quality of life (mean score on the FACT-L scale, range 0–136; higher scores indicate a better quality of life: 98.0 versus 91.5; *P*=0.03).^23^ Most respondents indicated that they would prefer to die at home in the presence of loved ones^23^; however, only one-third of patients in the United States are afforded this.^8^ Despite the clear benefits of these programs, public and healthcare providers were evidently poorly educated regarding them, which led to this unfortunate outcome.

### Barriers to Palliative Care

While the benefits of PC are numerous, there are many barriers to accessing these programs. A growing body of evidence has suggested that older people are more likely to be referred late to PC programs since referral is needs-based.^24^ The idea that elderly people have fewer requirements for PC is thought to be a consequence of death being more expected and potentially easier to come to terms with in this population.^24^ This belief leads to reduced utilization of specialized PC services.^24^ As the population ages, there will continue to be a growing need for PC programs. This increases the demand for improved home and community programs, as many people in need of PC live and prefer to stay at home, or reside in long-term care settings.^1^,^23^

The variability in disease trajectory and uncertainty of prognosis makes it difficult to identify patients that may benefit from PC. For example, in COPD patients, mortality is variable which makes it challenging for clinicians to recognize the transition to end of life.^25^ The existing model of prognostication is seen in Table 3 and comprises early and late stages of advanced cancer.^26^ The difficulties of using this existing model derive from the limited research into its application for non-malignant disease, and its reliance on physician estimation, which is subject to non-reproducibility.^27^ Given the complexity of this type of prognostic tool, an alternative screening tool, the “Surprise Question (SQ),” is routinely used to identify people who might benefit from PC services nearing the end of life.^28^ Studies have identified three triggers that may be utilized to initiate PC: the SQ, general indicators of decline, and specific clinical indicators related to certain conditions. The SQ helps physicians harness the clinical impression to plan the most appropriate care for each patient. This is done by asking questions such as: “Would you be surprised if the patient were to die in the next few months?” The utility of the SQ has been validated as a predictor for patients who are approaching the end of life, and it may simultaneously serve as a prognostic tool to improve the delivery of PC in the emergency setting.^29^ The SQ has also been confirmed to be an effective approach to screening patients who might need PC. However, further research and development are required to increase its effectiveness in conjunction with other parameters.^30^ If the answer to SQ is negative or uncertain, healthcare providers would then investigate and assess the general indicators of decline, delineated in Box 2.^31^,^32^ Decreasing response to treatment, repeated unplanned hospital admission, general physical decline, and declining functional performance status all suggest that PC may be a useful option for future management. The Palliative Performance Scale (PPS) serves to measure the progress of a patient’s general decline and is a guide to initiate and facilitate conversation about PC and end-of-life transition.^33^

**Table 3:** The Existing Model of Prognosis Between Early and Advanced Stages of Cancer.

Variable	Early Stage	Advanced Stage (Late Stage)
Prognosis	Years, decades	Months, weeks, days
Prognostic Factors	Clinical: stage, laboratory studies Pathological: histology, grade Molecular: gene, microarray Others: treatments, resources	Clinical: symptoms, laboratory studies Pathological: NA Molecular: NA Others: treatments, resources
Tools	Scores: International Prognostic Index Programs: Adjuvant online	Scores: Palliative Prognostic Score, Palliative Prognostic Index Programs: Palliative Performance scale
Implications	Overall outlook on life span Worse prognosis indicating more intensive cancer treatment New cancer treatment indicating changes in prognostic factors	End-of-life planning Worse prognosis; limit cancer treatment Limited cancer treatment; same prognostic factors

Box 2 General Indicators of Decline and Increasing Needs Among Patients.*

✓ Progressive disease and worsening symptoms
✓ Not responding to treatment or no further disease-modifying treatment available
✓ Declining functional performance status (inability to perform activities of daily living, Palliative Performance Scale <60)
✓ Many comorbid conditions
✓ Unintentional weight loss
✓ Frequent hospitalizations
✓ Sentinel event (falls, bereavement, etc.)

*Modified from Mississauga Halton Regional Hospice Palliative Care: Early Identification & Prognostic Indicator Guide^30^ and Thomas et al.^31^

Lack of basic training in PC for healthcare providers, as well as inadequate public knowledge, are the most frequently identified barriers to incorporating PC into treatment options.^23^,^33^ This issue is caused by a lack of education combined with differing views about the sanctity of life. As a result, emphasis on and opinions regarding use of aggressive therapies vary, all of which may negatively impact end-of-life quality. 35 Additionally, the tendency to associate PC with end-of-life and hospice care remains a barrier to the timely integration of PC and results from a lack of relevant knowledge. Conflicting treatment plans in PC occur when the treatment for the chronic illness stands in the way of the PC treatment or vice versa. Based on a 2013 public opinion survey, 90% of respondents agreed PC should be integrated into care for all people with chronic, life-limiting conditions, and 87% of them indicated that it should be available early in the course of a disease.^10^ Lastly, ineffective communication between healthcare providers during times of transition has also been categorized as a significant barrier to PC.^36^ Clinical work has been shown to suffer due to lack of coordination between staff in treatment of patients, with many citing lack of face-to-face contact and outdated electronic medical records technology as factors.^37^ The identified barriers to PC are summarized in Table 4.

**Table 4:** Identified Barriers to Palliative Care.

Barrier	References
Late referral associated with elderly patients	24
Lack of precision in the prognostication process	25–30,33
Lack of training and deficiency of PC knowledge	24,34,35
Poor communication between healthcare providers	36
Lack of public awareness of the role of PC	8,20
Stigma surrounding PC and its association with end of life	8

### Multidisciplinary Approach of Palliative Care

Multidisciplinary care is defined as a team approach to health care where healthcare professionals consider all relevant treatment options in a complicated illness with the goal to improve communication between patients, caregiver, and healthcare providers and help with coordination of care.^38^ The multidisciplinary approach utilized in PC is notable in the literature for a number of reasons. The role of PC at the end of life is to relieve the suffering of both patients and caregivers via well-established assessments and treatments. Palliative care does not merely help patients, it also provides support to the patient’s family during bereavement.^39^ Moreover, research by Silbermann illustrates the importance of the multidisciplinary team in offering impactful healthcare service to patients and their families.^40^

Nurses and social workers are in an ideal position to communicate patients’ social and emotional needs to other care providers if a relationship is developed early in the disease trajectory. Social workers play a crucial role in family care and crisis management in the different stages of grief, making them an essential component of a PC team. Social workers’ extensive knowledge of social support networks and financial assistance programs further enhances their importance in PC programs.^41^ Other allied healthcare providers including speech–language pathologists, physical therapists, occupational therapists, and registered dietitians all play an important role in maximizing patient quality of life and achieving the best possible comfort level at the end stages of terminal illness. The speech–language pathologists and registered dietitians are often in charge of developing strategies to maintain comfort feeding when there is a high risk of an aspiration event. They also support the patient in their ability to participate in decision-making with respect to the desired care.^42^ Furthermore, physical therapists and occupational therapists often play an important role in supporting patients by maintaining and fostering a sense of cohesion and independence by enhancing their mobility and occupational pursuits. In the PC environment, physical therapists and occupational therapists focus on “reverse rehabilitation” instead of making progress towards life, thus quality of life and comfort are insured until death. This is done by trying to incorporate daily activities into their treatment with the goal to potentially improve the quality of the remaining life.^43^ Lastly, the involvement of registered dietitians often exerts a positive influence in nutritional assessment. This subsequently leads to a better sense of comfort for the patient and higher family satisfaction level.^44^ Overall, multidisciplinary care aims to follow evidence-based guidelines and provides opportunities for both educational and quality assurance, which are aligned with the central aim of the PC program.^45^

### Early Referral to Palliative Care

The greatest benefits from PC programs are achieved when there is early integration and coordination of care. Compelling evidence has shown that early implementation of PC improves symptoms, quality of life, and disease outcomes for cancer patients when coupled with standard treatments.^19^ A study by Rowland highlighted the need for early referral and demonstrated no significant improvement in quality of life between “late” PC referral group and control group.^46^ Another study regarding early integration of PC for patients with advanced cancers demonstrated improvement in quality of life with no significant burden or cost to the patients.^47^ Furthermore, integration of PC has been proposed to foster improved service coordination, efficiency, and end-of-life quality for patients and family members.^48^ From patients’ perspectives, integrated PC underpins the importance of minimizing steps in the healthcare delivery and programs. Combined with the multidisciplinary approach, early integration of PC led to a better quality of life as evidenced in the study by Ellis among lung cancer patients.^45^

### Care Coordination of Palliative Care

Coordinating care around the individual is essential in maximizing the quality of end-of-life care. It ensures people receive the right care at the right time and in the right place. It helps patients to have a greater autonomy and control over their care with fewer unwanted hospital admissions. The Institute of Medicine in Duarte California identified care coordination as the pivotal step in improving the safety and effectiveness of the chosen care program. A study conducted by Dr Zachariah, for example, has shown that care coordination models, with early integration of PC, indeed lead to improvement among bladder cancer patients.^49^ Specifically, PC should be coordinated differently in the different environments such as in the community or a hospital setting. In the community setting, including patient homes and nursing homes, PC focuses on provision through established delivery systems such as home care. In this setting, nurses and social workers play a crucial role in coordinating and overseeing care in the community to maximize quality of life with consideration of the patient’s goals and preferences.^50^ A study conducted by Vimalananda et al. found that the most helpful contacts for patients were usually non-physician staff such as nurses.^51^ This is due to them being easier to contact and them being typically the first ones to notice any problems in treatment; however, their roles need to be centralized to ensure adequate patient care.^37^ Inpatient PC units are designed to support patients when they can no longer be cared for at home or within their community. Palliative care in the hospital setting is provided by a team ranging from primary care providers to PC consultants and other relevant specialists for the illness. These PC providers include physicians, nurses, pharmacists, and chaplains.^16^

Specialist PC doctors are in charge of managing the patient’s medical care and coordinating the care with other health professionals. The role of family physicians is to assist in coordination of care with specialists and act as generalist to support the consultation and transfer of care in ensuring the delivery of the standard clinical practice.^30^ Palliative care consultants mostly focus on dealing with complex cases and providing support and care for patients in acute care or long-term settings. Hospital-based PC consultation services have demonstrated improved physical and psychological symptom management, caregiver well-being, and markedly superior overall patient and family satisfaction.^52^

Traditionally, an interdisciplinary consultation team was made up of a doctor, a nurse, and a social worker. The new service-delivery or co-management model incorporates an interdisciplinary approach with more than one clinician. For example, with cancer patients, the PC clinician and oncologist will work together to treat the patients by dividing the tasks.^53^ The specific method and setting for end-of-life care depends on many factors. The type of disease plays a role in the type of treatment. Cancer has more of a predictable trajectory than non-malignant diseases like CHF; PC for CHF patients involves cardiologists along with the other PC providers.^16^ However, cardiologists often do not discuss PC until very late in the disease trajectory, creating a dilemma with respect to who is responsible for care coordination.^54^ Hence, it is important to identify better strategies to coordinate care between specialists, general practitioners, and PC providers in order to overcome the existing communication and implementation barriers related to PC.^17^ Professionals experienced difficulty communicating with each other in order to coordinate the delivery of care, with a reluctance to share information.^35^ Some general practitioners were reluctant to suggest that patients had PC needs.^35^ This stemmed from uncertainty among the staff regarding who the correct party was to communicate with, and what kind of information needed to be shared.^35^ Within the community setting, most end-of-life PC was provided by primary healthcare teams (PHCTs); this has proved satisfactory to patients with cancer-related treatment needs.^55^

Specialists, general practitioners, and nurses play a crucial role in the early identification of patients eligible for PC. This initiative has been shown to improve the quality of life care and maximize hospital resource utilization.^56^ It is also promising that the use of the electronic health record check can securely transmit patient data among healthcare providers, which ultimately enhances the coordination of PC among those with multiple chronic conditions. With respect to hospital settings, PC programs have grown by more than 150% over the past decade.^57^ Emergent or unplanned care is often provided by emergency medical services or professionals who are not connected to the patient’s ongoing healthcare management. This results in unnecessary costs, tests, increased medical errors, and a lack of communication and coordination between care teams and settings.^16^

Lastly, psychiatrists often have a person-centered approach, which is complemented by effective communication and supportive care for patients at different stages of their lives.^53^,^58^ Initiating PC is often thought of as a death sentence to patients and their families. Thus, discussing PC throughout primary treatment and involving psychiatrists may provide additional support and improve patients’ quality of life.^16^

## CONCLUSION

Although PC has traditionally been seen as a last resort, it is now being embraced as a necessary treatment in the early stages of both malignant and non-malignant chronic life-limiting illnesses. Education and open discussion play essential roles in the early integration of PC into treatment options. The growing need for improved access and equity in PC programs results from both an aging population and the widespread nature of chronic illnesses. Since the purpose of PC is to diminish suffering associated with life-threatening illnesses, it is imperative to advocate the associated benefits to the public and to healthcare providers.

## FUTURE DIRECTIONS

In addition to summarizing the previous research on PC for improving end-of-life care, the findings from this narrative review highlight several potential avenues for future research. Healthcare professionals must enhance their PC knowledge to better meet patients’ needs. The core competencies of PC, including communication and symptom management, have not received wide attention in most medical school training programs. Improved faculty capability in modeling and teaching state-of-the-art PC in academic health centers has been proposed to meet the teaching needs.^59^ Moreover, the PC needs of older adults with multiple coexisting conditions have yet to be well described. Better understanding of the needs of this patient population and their caregivers is required to develop a well-established PC model and allocate the specialist-level PC workforce efficiently. Lastly, a better transition to PC will ensure its coordination and continuity. The newly emerging concept of the MIH aims to facilitate the coordination of PC by closing unaddressed gaps (i.e. access to transportation, declining functional status, community support). Another type of gap the MIH model aims to close is poor care coordination causing failure to transmit patient information, harmful drug interactions, and conflicting treatment plans that may be presented by primary care providers or specialists.^60^

## Abbreviations

CHF: congestive heart failure
COPD: chronic obstructive pulmonary disease
EoLCS: End of Life Care Strategy
ESRD: end-stage renal disease
MIH: mobile integrated healthcare
NYHA: New York Heart Association
PC: palliative care
PCIP: Palliative Care Integration Project
PPS: Palliative Performance Scale
PRISMA: Preferred Reporting Items for Systematic Reviews and Meta-Analysis
RCTs: randomized control trials
WHO: World Health Organization
